# Piceatannol Is Superior to Resveratrol at Suppressing Adipogenesis in Human Visceral Adipose-Derived Stem Cells

**DOI:** 10.3390/plants10020366

**Published:** 2021-02-14

**Authors:** In Sil Park, Youngjin Han, HyunA Jo, Ki Won Lee, Yong Sang Song

**Affiliations:** 1 Department of Agricultural Biotechnology, Seoul National University, Seoul 08826, Korea; insil@snu.ac.kr (I.S.P.); youngjin.han@snu.ac.kr (Y.H.); whgusdk25@snu.ac.kr (H.J.); 2 Advanced Institute of Convergence Technology, Seoul National University, Suwon 16229, Korea; 3 Bio-MAX Institute, Seoul National University, Seoul 08826, Korea; 4 Research & Development Center, BOBSNU Co., Ltd., Suwon 16229, Korea; 5 Department of Obstetrics and Gynecology, Seoul National University College of Medicine, Seoul 03080, Korea

**Keywords:** resveratrol, piceatannol, obesity, adipogenesis, human visceral adipose-derived stem cells

## Abstract

Resveratrol (3,4′,5-trans-trihydroxystilbene) and piceatannol (3,3′,4′,5-trans-tetraphydroxystilbene) are major stilbene compounds that are predominantly present in various natural foods, such as berries and fruits. Both phytochemical compounds are consumed as dietary supplements to prevent various metabolic diseases and for their anti-aging properties. Adipose-derived stem cells from human visceral adipose tissue (vASCs) are a useful in vitro model for evaluating their adipogenic effect. Treatment with resveratrol and piceatannol significantly inhibited lipid accumulation in vASCs. Their effective concentrations were 5, 10, and 20 μM for inhibiting adipogenesis of vASCs. Interestingly, despite the similar chemical structures of the two compounds, piceatannol showed a higher anti-adipogenic effect at 20 μM than resveratrol in vASCs. Moreover, the inhibitory capacity of lipid droplet generation was higher for piceatannol at 20 μM than that of resveratrol. Piceatannol significantly attenuated the expression level of adipogenic markers (e.g., CCAAT/enhanced binding protein α (C/EBPα), peroxisome proliferator-activated receptor γ (PPARγ), and adipocyte fatty acid binding protein (aP2)) compared to resveratrol at the mRNA and protein levels. These results suggest that piceatannol is a superior anti-adipogenic compound compared to resveratrol in the vASC model of visceral obesity.

## 1. Introduction

Resveratrol (3,4′,5-trans-trihydroxystilbene, Res) and piceatannol (3,3′,4′,5-trans- tetraphydroxystilbene, Pic) are polyphenolic compounds. Res was first isolated in 1940 from the roots of white hellebore (*Veratrum grandiflorum O. Loes*) and the disease prevention effects of Res have since been extensively reported. Res has beneficial effects on many disease types, such as neurological disorders [1], cardiovascular disease [2,3], and cancer [4,5]. The identification and quantification of Pic were initially reported from the analysis of the domesticated oilseed, *Euphorbia lagascae* [6]. Although the health-promoting effects of Pic has been less extensively studied compared to Res, Pic has demonstrated cardiovascular disease prevention effects [7], anti-cancer effects [8,9], and anti-inflammatory activity [10].

**Figure 1:**
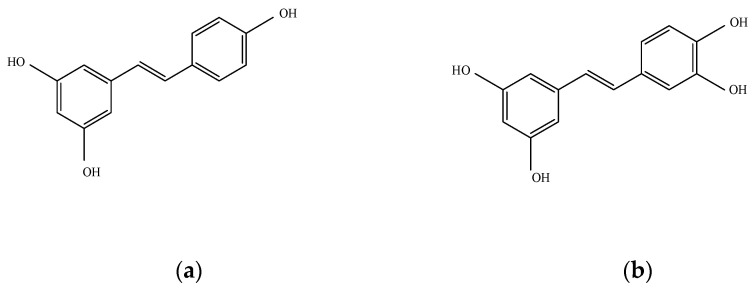
The chemical structure of (**a**) resveratrol (Res) and (**b**) piceatannol (Pic). In the structure formula of Res, two aromatic rings are connected by one methylene double bond. Pic is a hydroxyl derivative of Res.

Res and Pic are major stilbene compounds that are predominantly present in various natural products, such as sim fruit (*Rhodomyrtus tomentosa*) seeds [11] and passion fruit (*Passiflora edulis*) seeds [12]. The molecular structure of Res comprises two aromatic rings connected by a methylene double bond. The chemical structures of Res and Pic are shown in Figure 1a,b. Res and Pic have similar chemical structures, but Pic is a 3′hydroxylated analog of Res (Figure 1a,b). Owing to the similarity in chemical structure, both Res and Pic possess similar biological activities, including antioxidants [13], decreasing tumor cell survival in colon cancer [14], and neuroprotective activity [15].

Among the various disorders, the prevalence of obesity tripled from 1975 to 2016. According to the World Health Organization (WHO), 39% of adults are overweight or obese, thereby implying that obesity is becoming a pandemic [16]. Obesity is considered a significant risk factor for cardiovascular disease, diabetes, and cancer leading to increased mortality worldwide [17,18]. Obesity originates from excessive lipid accumulation in adipose tissue. An increase in adipose tissue proportions is characterized by an increase in adipocyte cell number and size. Adipocytes are derived from mesenchymal stem cells [19,20]. Adipogenesis is tightly regulated by key adipogenic transcription factors, including the members of CCAAT/enhanced binding protein (C/EBP) and peroxisome proliferator-activated receptor γ (PPARγ). Members of the C/EBP family include C/EBPα, C/EBPβ, and C/EBPγ. Among them, C/EBPα is mainly involved in the cell fate during adipocyte differentiation. In addition, PPARγ is required for adipocyte differentiation and maintenance of mature adipocytes. Furthermore, the expression of adipocyte fatty acid binding protein 2 (aP2) is altered during adipogenesis [21]. To identify natural food compounds that can efficiently prevent obesity, various natural compounds have been investigated for their anti-adipogenic effects.

In terms of the adipogenic effects of Res and Pic, anti-adipogenic effects have been observed in various in vitro models. Treatment with Res inhibits the maturation of 3T3-L1 cells through the modulation of the insulin signaling pathways present in the murine preadipocyte cell line 3T3-L1 [22,23]. Pic inhibits the early phase of differentiation from 3T3-L1 to adipocytes by regulating the mitotic clonal expansion and insulin signaling [24]. Moreover, a similar anti-adipogenic effect occurs in adipose-derived stem cells (ASCs) from human subcutaneous adipose tissue (sASCs) [25]. Although previous studies have utilized these cell lines and sASCs as useful models for studying the anti-adipogenic effects of Res and Pic, their effects on ASCs from human visceral adipose tissue (vASCs) have not yet been evaluated. Furthermore, previous studies have shown transcriptomic disparities between vASCs and sASCs, as well as human ASCs and 3T3-L1 cells [26]. Therefore, the effect of Res and Pic on adipogenesis could be different depending on the stem cells from different tissues of origin [27]. More importantly, the accumulation of visceral fat is closely associated with numerous metabolic disorders [18]. Therefore, there is an increasing demand to study the effects of Res and Pic using vASCs.

Pic is more potent than Res in promoting health. For instance, the generation of astrocytes has a central role in brain development, and the astrocyte differentiation capacity in adult mouse brains from neural stem cells of Pic is higher than that of Res, implying that Pic improves brain functions to a large degree [28]. Pic has greater metabolic stability than Res when the absorption and metabolism of both are compared [29]. In addition, Pic activates sirtuin 1 (SIRT1) expression in THP-1 monocytic cells to a greater degree than Res [30]. As these two compounds with similar chemical structures are present in the dietary supplements, it is essential to identify which compound has better anti-adipogenic capacity.

The present study was performed to investigate whether Res and Pic attenuate adipogenesis in human vASCs and to compared their anti-adipogenic activity.

## 2. Results

### 2.1. The Effect of RES and Pic on Cell Viability of vASCs

**Figure 2:**
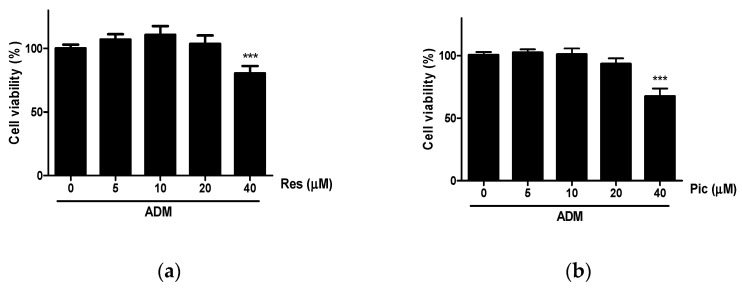
The effect of resveratrol (Res) (**a**) and piceatannol (Pic) (**b**) on the cell viability of human visceral adipose-derived stem cells (vASCs). vASCs were treated with the Res and Pic in the presence of adipogenic differentiation medium (ADM) for two days. Cell viability was quantified using the MTT assay and absorbance was measured at 570 nm. Data are expressed as means ± SD from three independent experiments (*** *p* < 0.001 vs. ADM-treated-group).

A 3-(4,5-dimethylthiazol-2-yl)-2,5-diphenyltetrazolium bromide (MTT) assay was conducted to investigate the cytotoxicity of Res and Pic in vASCs cultured with adipogenic differentiation medium (ADM) for two days and treated at concentrations ranging from 0–40 μΜ. When up to 20 μΜ Res and Pic were applied to vASCs, cytotoxicity in vASCs was not observed. However, in the 40 μΜ Res-treated group, cell viability decreased by 20% compared to the ADM-only-treated group. In the vASC group treated with 40 μΜ of Pic, the cell viability decreased by 33% compared to the ADM-only-treated group (Figure 2a,b). Therefore, the maximum tolerable concentration of Res and Pic in vASCs for evaluating the adipogenic effect was up to 20 μΜ.

### 2.2. The Effect of Res and Pic on the Lipid Accumulation of vASCs

**Figure 3:**
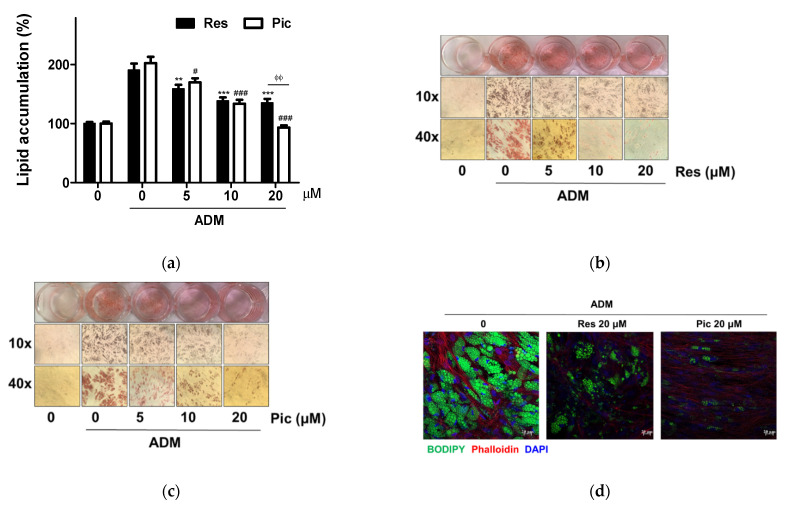
(**a**) Human visceral adipose-derived stem cells (vASCs) were treated with the resveratrol (Res) and piceatannol (Pic) in the absence or presence of adipogenic differentiation medium (ADM) for 14 days and stained with oil red O (ORO). Intracellular lipid accumulation levels were quantified by extracting ORO-stained lipid droplets with 100% isopropanol and the absorbance was measured at 495 nm. Data are expressed as means ± SD from three independent experiments (** *p* < 0.01 and *** *p* < 0.001 vs. ADM-only-treated group of the Res treatment groups; ^#^ *p* < 0.05 and ^###^ *p* < 0.001 vs. ADM-only-treated group of the Pic treatment groups; ^ᶲᶲ^ *p* < 0.01). (**b**,**c**) Photographs are representative images of the three independent experiments (magnification 10× and 40×). (**d**) vASCs were treated with Res and Pic in the presence of ADM for 14 days and stained with 4,4-difluoro-4-bora-3a,4a-diaza-s-indacene (BODIPY). Confocal images of fat body stained with BODIPY 493/503, Phalloidin, and 4′,6-diamidino-2-phenylindole (DAPI) to show lipid droplets, actin filaments, and nuclei, respectively.

To evaluate the anti-adipogenic effect of Res and Pic, vASCs were differentiated with ADM after their application at concentrations up to 20 μM for 14 days. Intracellular lipid content was assessed using Oil Red O (ORO) staining. ADM treatment significantly increased the lipid accumulation of vASCs. However, treatment with Res on vASCs reduced ADM-induced intracellular lipid accumulation by 17% (*p* < 0.01), 27% (*p* < 0.001), and 29% (*p* < 0.001) after treatment with 5, 10, and 20 μM, respectively. In addition, Pic-treated vASCs also decreased ADM-induced intracellular lipid accumulation by 16% (*p* < 0.05), 34% (*p* < 0.001), and 54% (*p* < 0.001) after treatment with 5, 10, and 20 μM, respectively. Both Res and Pic showed substantial inhibition of vASCs adipogenesis. Interestingly, when Res and Pic were administered at 20 μM, the Pic-treated group showed 31% (*p* < 0.01) higher anti-adipogenic capacity than that in the Res-treated group (Figure 3a). Therefore, Pic had a higher inhibitory effect in vASCs than Res. Our results showed that both Res and Pic exerted a significant anti-adipogenic effect in vASCs at concentrations ranging from 5 to 20 μM (Figure 3a–c). To assess the role of Res and Pic treatment on the adipogenesis of vASCs, lipid droplets were stained with 4,4-difluoro-4-bora-3a,4a-diaza-s-indacene (BODIPY) in Res- and Pic-treated vASCs after 14 days of incubation. Confocal microscope images showed the lipid (green channel), nucleus (blue channel), and actin filaments (red channel). The decrease in the number of lipid droplets was greater in the Res-treated group than in the Pic-treated group under adipogenic conditions (Figure 3d).

### 2.3. Comparison of Res and Pic on the Modulation of Adipogenic Marker Expression in vASCs

**Figure 4:**
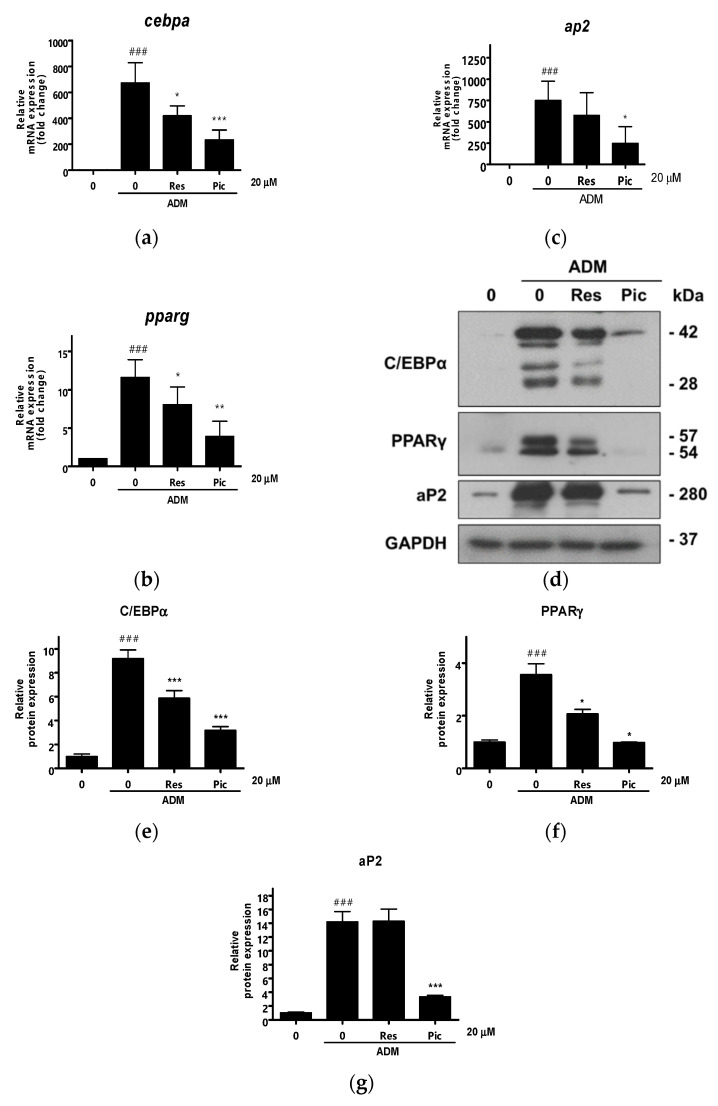
The effect of resveratrol (Res) and piceatannol (Pic) on the expression of adipogenic differentiation markers in human visceral adipose-derived stem cells (vASCs). (**a**–**c**) The relative mRNA expression of adipogenic genes, namely CCAAT/enhanced binding protein α (C/EBPα), peroxisome proliferator-activated receptor γ (PPARγ), and adipocyte fatty acid binding protein (aP2), were analyzed using quantitative reverse transcription polymerase chain reaction (qRT-PCR) on day three after adipogenic differentiation medium (ADM) treatment. Data are expressed as means ± SD from three independent experiments (^###^ *p* < 0.001 vs. undifferentiated group; * *p* < 0.05, ** *p* < 0.01, and *** *p* < 0.001 vs. ADM-treated group). (**d**–**g**) The protein expression of adipogenesis related proteins, namely CEBP/α, PPARγ, and aP2, were analyzed using Western blot on day four after ADM treatment. Data are expressed as means ± SD from three independent experiments (### *p* < 0.001 vs. undifferentiated group; * *p* < 0.05 and *** *p* < 0.001 vs. ADM treated group).

Next, quantitative reverse transcription-polymerase chain reaction (qRT-PCR) was performed to determine whether Res and Pic affected the expression of adipogenic transcription factors. The transcript expression of vASCs was examined three days after ADM treatment in vASCs with or without Res or Pic treatment. The mRNA levels of key adipogenesis genes, namely C/EBPα, PPARγ, and aP2, were significantly increased by ADM treatment in vASCs. In contrast, in the 20 μM of Res- and Pic- treated group, the mRNA levels of C/EBPα, PPARγ, and aP2 significantly decreased compared to those in the ADM-only treated group. Moreover, when comparing the Res and Pic treatment groups, the reduction in mRNA expression of C/EBPα, PPARγ, and aP2 was higher in the Pic-treated group than in the Res-treated group (Figure 4a–c). Consistently, the protein expression levels of C/EBPα, PPARγ, and aP2 were markedly upregulated by ADM treatment, and the increase in the adipogenic marker expression at the protein level was reversed by the addition of Res and Pic to vASCs. In addition, in the presence of Pic, the expression levels of C/EBPα, PPARγ, and aP2 were higher in the Res-treated group than in the Pic-treated group (Figure 4d–g). Taken together, we concluded that Pic is more effective at inhibiting adipocyte differentiation in vASCs than Res.

## 3. Discussion

In the present study, Res and Pic treatment showed a significant decrease in intracellular lipid accumulation in vASCs. However, Pic showed a higher inhibitory effect than Res on the anti-adipogenesis of vASCs. Therefore, a natural product rich in Res and Pic would have the effect of inhibiting vASC differentiation into adipocytes. Furthermore, we suggest that Pic is a more effective anti-adipogenic compound than Res.

The maximum tolerable concentration of Res and Pic was evaluated on vASCs using the MTT assay. When Res and Pic were applied to vASCs at up to 40 μΜ, decreased cell viability was observed at 40 μΜ. Therefore, vASCs were treated with Res or Pic to evaluate the adipogenic effects of concentrations up to 20 μΜ (Figure 2a,b). In a previous study, up to 40 μΜ of both Res and Pic in β-amyloid (Aβ)-induced rat primary cerebral cortex neurons did not decrease cell viability compared with that of the Aβ-only treatment group [15]. In contrast, Res and Pic showed cell cytotoxicity at 40 μΜ in our study. These disparities in cytotoxicity may be due to the different origins and natures of cells.

Treatment with Res and Pic in vASCs reduced intracellular lipid accumulation when treated at 5, 10, and 20 μΜ. The anti-adipogenic effect of Res and Pic treatment in vASCs was consistent with those of previous studies evaluating the adipogenic effects of Res and Pic. Both Res and Pic inhibited the differentiation of mature adipocytes. Treatment with Res inhibited adipogenesis in 3T3-L1 cells at 20 and 100 μM [22,23]. Pic treatment also showed an inhibitory effect on the adipogenesis of 3T3-L1 cells at 25 and 50 μM [24], and sASCs at 50 μM [25]. In contrast, treatment with Res increased adipocyte differentiation at 1 and 10 μM in 3T3-L1 cells. Res treatment showed different efficacy, which was thought to be because of the effect of Res on adipocytes was mediated by multiple targets. In addition, Res has been investigated for the promotion of osteogenic differentiation in sASCs [31] and mesenchymal stem cells isolated from canine adipose tissue [32]. Several in vitro studies have demonstrated that the factors associated with lipid accumulation inhibit bone mineralization, whereas those related to osteoblast differentiation suppress adipocyte differentiation [33]. Therefore, our results, which showed that Res inhibits adipocyte differentiation in vASCs, is consistent with those that promote effects on osteoblast differentiation by Res treatment due to the adipo-osteogenic balance being controlled during mesenchymal stem cells differentiation.

Pic had a higher inhibitory effect on lipid deposition in vASCs than Res. This might be a consequence of the different molecular structures of Res and Pic. Pic has an additional 3,4-hydroxyl structure on a benzene ring compared to Res (Figure 1a,b). Pic possesses a higher radical scavenging capacity than Res since the increased number of hydroxyl groups in the structure causes scavenging of reactive oxygen species (ROS) and stabilizing of phenolic oxygen radicals [34]. Previously, the adipogenic capacity of adipose precursors isolated from the visceral fat of high-fat diet (HFD) mice was related to increased ROS levels [35]. In addition, the enhanced antioxidative effect of Pic has a protective effect in acute cardiac injury [36]. Therefore, the chemical structure of Pic, which differs from that of Res, could be an important factor for its anti-adipogenic activity. Scavenging intracellular ROS in vASCs could be a critical approach for inhibiting the adipogenesis of vASCs. Further investigation regarding the mechanisms associated with the differential adipogenic effects of Res and Pic in vASCs is needed. The molecular mechanisms of Res and Pic on the anti-adipogenic effect have been previously discussed. Res treatment inhibits adipogenesis by including SIRT1 dependent apoptosis by upregulating adenosine-monophosphate-activated protein kinase (AMPK) and downregulating protein kinase B (AKT) activity in 3T3-L1 cells [22,23]. Furthermore, the addition of Pic to 3T3-L1 downregulates the insulin receptor-dependent signaling pathway and inhibits phosphoinositide 3-kinase (PI3K) and AKT activity [24]. Therefore, the anti-adipogenic effect of Res and Pic in vASCs may regulate the SIRT1, AMPK, and AKT signaling pathways, and the expression levels of SIRT1, AMPK, and AKT may show different levels between Res and Pic treatment. Increasing evidence suggests that nutraceuticals from natural products attenuate adipogenesis in mesenchymal stem cells by activating the wingless-type mouse mammary tumor virus (MMTV) integration site (WNT)/β-catenin signaling pathways [37,38]. Therefore, it would be interesting to study the underlying molecular mechanisms involved in regulating the adipogenesis of vASCs by Res and Pic.

A clinical study showed that Res supplementation has significant body weight-lowering effects in obese adults [39]. However, another study showed that there was no significant change in the body weight in obese adults when supplemented with Res [40]. The mixed results of clinical trials using Res suggest that future studies on the therapeutic potential of Pic and Res could be performed in obese adults through a comparative study on the inhibitory effect of Res and Pic on adipogenesis. According to our results, Pic as a dietary supplement prevents obesity, and it may have more significant potential than Res in the management of obesity. In a pharmacokinetic study of intragastric administration of Res and Pic in rats, the area under the plasma concentration curves representing bioavailability was 2.1 times higher in Pic than in Res. Therefore, Pic had a higher absorption and a higher metabolic stability than Res [28]. Moreover, a recent study reported that Pic converted to isorhapontigenin (Iso) in rats after oral administration [41]. Iso possesses a higher oral bioavailability and anti-inflammatory effects in chronic obstructive pulmonary disease than Res [42].

The weight loss effect of Pic in a HFD mouse model has been previously reported. It was demonstrated that the weight of perigonadal and retroperitoneal fat, which is equivalent to the visceral depot in the human body, was also decreased by Pic supplementation in a HFD-induced obesity mouse model [43]. Although the anti-visceral obesity effect of Pic has been previously shown in the HFD mouse model, the impact of Pic on body weight and visceral fat in humans is, to the best of our knowledge, yet to be determined. Mouse models are commonly used to predict human disease and drug responses. However, results often fail to translate to the corresponding human disease due to differences in species, drug metabolism, and gene expression profiles [44,45]. For instance, Res has been reported to decrease body weight and intra-abdominal fat tissue in the HFD mouse model [46]. However, in a clinical study investigating the weight loss effects of Res on 24 obese adults, supplementation with Res (500 mg per day for 4 weeks) did not show any effects on visceral fat weight [40]. Our study showed that Pic possesses an anti-adipogenic effect in vASCs. Therefore, the novelty of this study is that vASCs were used as a model for evaluating and comparing the anti-adipogenic effects of Res and Pic.

Adipose tissue stores lipids through enlargement of existing adipocytes and recruitment of new adipocytes to accommodate excess lipid intake [47]. However, the inability of adipogenesis in adipocytes of adipose tissue leads to ectopic lipid accumulation in other tissues, such as the liver, skeletal muscle, and heart [48]. Pic showed decreasing adipogenesis in ASCs of visceral fat from our results. There is a possibility that Pic may increase the risk of ectopic fat accumulation and further studies are needed to verify this hypothesis.

## 4. Materials and Methods 

### 4.1. Reagents

Res and Pic were purchased from Sigma-Aldrich (St. Louis, MO, USA). The chemical structures of Res and Pic are shown in Figure 1. The anti-cluster of differentiation 31 (anti-CD31) and anti-cluster of differentiation 45 (anti-CD45) microbeads and magnetic cell sorting system (MACS) separation buffer were purchased from Miltenyl Biotec (Bergisch Galdbach, Germany). MesenPRO RS medium, GlutaMAX, and insulin were purchased from Gibco (Waltham, MA, USA). Collagenase type IA, 3-isobutyl-1-methylzanthine (IBMX), indomethacin, dexamethasone, and oil red O were purchased from Sigma-Aldrich (St. Louis, MO, USA). Trizol and reverse transcriptase were purchased from Takara (Kusatsu, Japan). SYBR supermix was purchased from Bio-Rad Laboratories (Hercules, CA, USA). Antibodies against C/EBPα (#2295), PPARγ (#2430), and aP2 (#2120) were purchased from Cell Signaling (Danvers, MA, USA). Antibodies against glyceraldehyde 3-phosphate dehydrogenase (GAPDH) (LF-PA0202) were purchased from Ab Frontier (Seoul, Korea). Four-well chamber slides with removable wells were purchased from Thermo Fisher Scientific (Waltham, MA, USA). Collagen I, rat tail (354236) was purchased from Corning (Corning, NY, USA). BODIPY 493/503 staining (D3922), Alexa Fluor 647 phalloidin (A22287), ProLong Glass antifade mountant (P36982), and DAPI (D1306) were purchased from Invitrogen (Carlsbad, CA, USA). 

### 4.2. Isolation of Human Visceral vASCs

Clinical information regarding the donors of vASCs is shown in Table 1. Visceral adipose tissue (VAT) was collected from the intra-abdomen of human donors (*n* = 5) during gynecologic surgery. The waist-to-hip ratio (WHR) is a strong predictor of visceral obesity [49]. Therefore, based on WHR, vASCs obtained from five donors with visceral obesity (WHR ≥ 0.85) were used. The procedure was approved by the Seoul National University Hospital Institutional Review Board (SNU-1003-009-311). This study was conducted in accordance with the Declaration of Helsinki and informed consent was obtained from the donors for this research. vASCs were isolated as described previously [4]. Briefly, blood vessels of the VAT were removed. VAT was dissociated with collagenase type IA and diluted in 0.25 mg/mL phosphate-buffered saline (PBS) for 1 h at 37 °C, and then centrifuged at 500× g for 4 min. After centrifugation, the stromal vascular fraction (SVF) pellet was collected. The SVF was filtered using MACS through a negative selection of CD31 (endothelial cell marker) and CD45 (hematopoietic stem cell marker). The CD31 and CD45 negative SVF were plated onto a 100 mm culture dish with MesenPRO RS medium containing 1% GlutaMAX and 1% penicillin-streptomycin (PS). After three days of vASCs seeding, non-adherent cells were removed via PBS washing. Expansion of vASCs was performed in a two-passage process before experimental use. vASCs were used for experiments in passages 3–6.

### 4.3. Differentiation of Adipocytes from vASCs 

vASCs were seeded on 48-well plates at a density of 0.03 × 10^6^ cells/well and cultured in MesenPRO RS medium. When vASCs reached more than 95% confluency, the cells were incubated with ADM. ADM was prepared by mixing 10% FBS, 1% PS, 10 μg/mL insulin, 0.5 mM IBMX, 50 μM indomethacin, and 1 μM dexamethasone in DMEM-F12. ADM was changed every second day, and the cells were incubated for 14 days. 

### 4.4. ORO Staining

Differentiation of vASCs into adipocytes was examined using ORO staining. Differentiated adipocytes were washed with PBS, and then fixed in 4% formaldehyde for 30 min. Then, the fixed cells were then washed with deionized water, and 60% saturated ORO staining was carried out for 1 h. For ORO quantification, isopropanol was added to each well. Light absorbance was measured at 495 nm.

### 4.5. BODIPY Staining

vASCs cells were seeded on 4-well chamber slides at a density of 0.01 × 10^6^ cells per well. After two days of incubation, vASCs were cultured in ADM with Res and Pic treatment for 14 days. The cells were washed and fixed with 4% formaldehyde for 15 min. Cells were incubated with BODIPY 493/503 and phalloidin for 2 h at 25 °C and then washed with 0.1% Tween20 in PBS/T. After washing, the cells were stained with DAPI and mounted. After mounting, the fluorescence signal was detected under confocal microscopy (LSM800, Zeiss). The maximum absorption wavelengths were 488 nm, 674 nm, and 350 nm for BODIPY, Phalloidin, and DAPI, respectively.

### 4.6. qRT-PCR

RNA of vASCs was extracted using Trizol reagent and RNA concentration was determined using Nanodrop (Nano Drip 2000, Thermo Scientific). cDNA was obtained using reverse transcription containing 1 μg of total RNA, oligo (dT), and reverse transcription premix. The qRT-PCR reactions were performed using the SYBR green PCR system (CFX96, Bio-Rad) in a thermal cycler (C1000, Bio-Rad). The cycling conditions were as follows: 95 °C for 10 min followed by 38 cycles involving denaturing at 95 °C for 5 s, annealing at 60 °C for 15 s, and an extension at 72 °C for 10 s. Expression of mRNAs were normalized to the mRNA levels of GAPDH. The forward and reverse primer sequences are listed in Table 2.

### 4.7. Western Blot 

vASCs were lysed with a lysis buffer supplemented with 1% Triton X-100, EDTA-free protease inhibitor cocktail, Na3VO4, phenyl methyl sulfonyl fluoride, and sodium deoxycholate. A bicinchoninic acid (BCA) protein assay was performed to measure protein concentrations. Protein samples (5 μg per well) in each sample were separated using 9% sodium dodecyl sulphate–polyacrylamide gel electrophoresis (SDS-PAGE) and transferred to a nitrocellulose membrane. The membrane was blocked with 5% skim milk in Tris-buffered saline containing 0.1% Tween 20 (TBS-T) and incubated with primary antibodies (1:1000 dilution) overnight at 4 °C. The membrane was incubated with a peroxidase-conjugated secondary antibody (1:10000 dilution). The blots were detected using a Western blot detection kit. 

### 4.8. Statistical Analysis 

Data are expressed as mean values ± standard deviation (SD) of three independent experiments. For multiple comparisons, analysis of variance was used followed by Tukey’s test. Statistical analysis was performed using SPSS (version 26). Student’s t-test was used for the comparison between two independent groups. Differences were regarded as significant if the value was *p* < 0.05.

## 5. Conclusions

Treatment with Res and Pic significantly inhibited of lipid accumulation in vASC concentrations ranging from 5 to 20 μM, where cell cytotoxicity was not observed. Interestingly, Pic showed a higher anti-adipogenic effect at 20 μM than Res in vASCs. Moreover, the inhibitory capacity of lipid droplet generation was higher for the Pic at the 20 μM concentration than that of Res. Pic more significantly attenuated the expression level of adipogenic markers (e.g., C/EBPα, PPARγ, and aP2) compared to Res at both the mRNA and protein levels. Our results suggest that Pic is a superior anti-adipogenic compound compared with Res in the vASC model of visceral obesity. 

## Figures and Tables

**Table 1:** Clinical information regarding the donors of vASCs.

Donor No.	Age	WHR	BMI
#31	66	0.98	23.94
#34	66	0.97	25.56
#47	71	0.98	24.36
#50	54	0.94	22.99
#54	68	1.04	27.37

WHR—waist-to-hip ratio; BMI—body mass index.

**Table 2:** Primer sequences used for mRNA expression.

Genes	Forward Primer (5′-3′)	Reverse Primer (5′-3′)
C/EBPα	GCAAACTCACCGCTCCAATG	CTTCTCTCATGGGGGTCTGC
PPARγ	AGGTCAGCGGACTCTGGATTC	AGTGGGGATGTCTCATAATG
aP2	ATGGGGGTGTCCTGGTACAT	ACGTCCCTTGGCTTATGCTC
GAPDH	GAGTCAACGGATTTGGTCGT	TTGATTTTGGAGGGATCTCG

## Data Availability

Not applicable.
